# Evaluation of the Sedative and Anesthetic Effects of *Protium heptaphyllum* Essential Oil in Juvenile *Colossoma macropomum* (Cuvier 1818): A Behavioral, Electrophysiological, and Plasma Glucose Study

**DOI:** 10.1021/acsomega.6c01086

**Published:** 2026-04-28

**Authors:** Luciana Eiró-Quirino, Axell Timotheo Lima Acioli Lins, Luana Vasconcelos de Souza, Alex Luiz Menezes da Silva, Marcele Fonseca Passos, Moisés Hamoy

**Affiliations:** † 37871 Laboratório de Farmacologia e Toxicologia de Produtos Naturais/ICB/UFPA, Belém 66073-110, Brazil; ‡ Institute of Health Sciences, Post - Graduation Program in Pharmaceutical Sciences, Biomaterials, Bioproducts, and Biomanufacturing Technologies Laboratory, Federal University of Pará, Belém 66079-420, Brazil

## Abstract

This study evaluated the sedative and anesthetic effects of *Protium heptaphyllum* essential oil (PHEO) in juvenile *Colossoma macropomum* through behavioral, electrophysiological, and biochemical analyses. Fish were exposed to different concentrations of PHEO (50, 75, 100, and 125 μL·L^–1^), and the parameters analyzed included latency to loss and recovery of postural reflex, cardiac activity (electrocardiogram), opercular movement, and plasma glucose levels. Chromatographic analysis revealed *p*-cymene, limonene, alpha-pinene and β-phellandrene as the major constituents of the oil. The results showed that PHEO induced sedation and anesthesia in a concentration-dependent manner, promoting bradycardia and a reduction in opercular movement frequency, with partial reversibility after exposure ceased. Plasma glucose increased significantly at higher concentrations. We conclude that PHEO has potential as a natural and safe anesthetic for aquaculture, especially at moderate concentrations.

## Graphical abstract

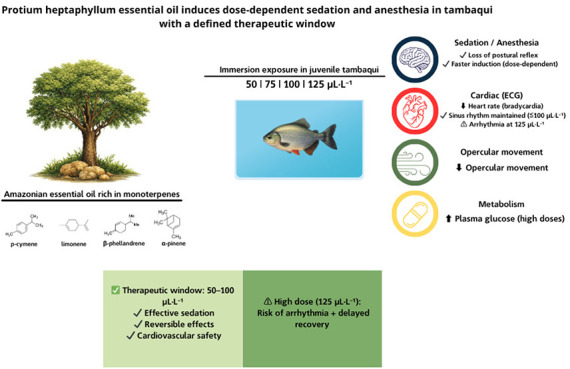

## 1 Introduction

Essential oils are natural, volatile, and complex substances, recognized for their intense aroma, derived from aromatic plants as secondary products of metabolism and are usually extracted by steam or hydrodistillation.^1^ In addition, they possess antiseptic and medicinal properties such as anxiolytic,^2^ analgesic,^3^ sedative,^4^ anti-inflammatory,^5^ spasmolytic,^6^ local anesthetic,^7^ and anticancer^8^ actions. They are also used in embalming due to their antimicrobial and antioxidant activity.^9^

The *Burseraceae* family encompasses 21 genera and 600 species, with the genus Protium being the most prominent, comprising 135 species.^10^ These plants are notable in ethnobotany and are used in traditional medicine for the treatment of wounds, control of inflammation, and as an insect repellent; in addition, their oleoresin has promising antimicrobial effects.^11^ Furthermore, regarding its chemical profile, the essential oil of *Protium heptaphyllum* resin presents *p*-cymene (39.93%) as its main component, which is an aromatic hydrocarbon with diverse functions.^12^

The tambaqui (*Colossoma macropomum*) is a tropical fish native to the Amazon and Orinoco river basins, belonging to the *Serrasalmidae* family, and has been expanding in aquaculture due to its rapid growth and adaptability.^13^ Advances in fish farming science have favored the production of tambaqui, a species that adapts to monoculture, polyculture systems, and extensive and intensive production methods, seeking greater efficiency with less environmental impact.^14^ During their life cycle in rearing, the fish undergo frequent handling and confinement, such as capture, weighing, vaccination, transport, and slaughter, and therefore, the use of anesthetics and sedatives is important to ensure their well-being during these processes.^15^

Anesthesia is a drug-induced state that results in the temporary loss of sensation and, in some cases, consciousness through the blocking of nerve impulses that transmit the perception of pain.^15^ Sedation is a decrease in sensitivity, leading to tranquility and serenity, with decreased locomotion, hypothermia, and hypnosis being indicative of hypnosedative effects.^16^

Manually monitoring the behavior of fish in cages is challenging but necessary to maintain a healthy environment, as stressed fish exhibit frantic movements caused by physical, chemical, or environmental factors, affecting the well-being of these animals.^17^ Furthermore, fish, like mammals, maintain a specific level of glucose in their blood, influenced by factors such as hormones, diet, and temperature, and therefore, blood glucose is a valuable parameter for researchers to assess the immediate condition of an organism.^18,19^

Chromatography in essential oils is essential for recognizing substances, verifying purity, and identifying contaminants.^20^ In this method, the diluted sample is transported through a column filled with a stationary phase that separates its compounds by different retention times, which subsequently allow the identification of the peaks corresponding to each compound, enabling its recognition and quantification.^21^

Given the above, it becomes essential to evaluate natural alternatives that can act as effective anesthetics in farmed fish, with lower ecotoxicological risk and controllable physiological effects.^34,35^ The essential oil of *Protium heptaphyllum* emerges as a promising candidate, considering its ethnopharmacological history and complex chemical profile. Thus, the present study aimed to investigate the sedative and anesthetic effects of *Protium heptaphyllum* essential oil (PHEO) in juvenile *Colossoma macropomum*, using an integrated approach that included behavioral, electrophysiological, and biochemical analyses, focusing on plasma glucose as a physiological marker.

## 2 Materials and Methods

### 2.1 Experimental Animals

The individuals used (*n* = 126) of the tambaqui species, *Colossoma macropomum*, (males and females), were stocked in aquariums at the Experimental Bioterium of the Laboratory of Pharmacology and Toxicology of Natural Products of the Federal University of Pará (ICB/UFPA) in an environment with controlled temperature (25 to 27 °C) and photoperiod 12 h C: 12 h E. Feeding was carried out twice a day with commercial feed (32% protein) until satiated. Concomitantly with siphoning to remove feces and uneaten food, the water was partially renewed (approximately 30% of the tank volume) with filtered water of the same origin. During acclimation (15 days), water quality variables such as water temperature (°C) (26.3 °C); pH (pH = 7.6) were monitored. For all experiments, *n* = 9 refers to nine animals per experimental group. Behavioral analysis, electrocardiographic recordings, opercular rate measurements, and blood glucose determination were performed using independent biological replicates within each treatment group.

### 2.2 Organoleptic Properties and Chromatographic Analysis

The essential oil of *Protium heptaphyllum* (PHEO) was obtained from Laszlo Aromatherapy Laboratory (Brazil; CNPJ 07.997.093/0001–10), which produces the oil from oleoresin, and extracted by steam-distillation. Chemical characterization was performed by high-performance gas chromatography using an Agilent 7820A gas chromatograph equipped with a flame ionization detector (FID) and an HP-5 capillary column (30 m × 0.32 mm i.d., 0.25 μm film thickness). The oven temperature was programmed from 70 °C (initial hold 0 min) to 250 °C at 3 °C min^–1^. The injector temperature was set at 250 °C, with a split ratio of 1:50. The FID temperature was maintained at 260 °C. A 1 μL aliquot of the sample (1% in chloroform) was injected. Analyses were performed at the Institute of Natural Products Research, Federal University of Rio de Janeiro (RJ, Brazil). Compound identification was based on retention time comparison and literature data, and relative percentages were calculated from peak areas without correction factors. The major constituents were *p*-cymene, limonene, and β-phellandrene (Table 1; Figure 1).

**Table 1:** Components Identified in the Essential Oil of *Protium heptaphyllum* by GC-MS and GC-DIC

#	Retention time (min.)	Kovats	Area (%)	Compound
1	5.67	928	0.15	Alpha-thujene
2	5.89	937	13.60	Alpha-pinene
3	6.35	954	0.34	Camphene
4	6.98	975	0.28	Sabinene
5	7.16	980	1,97	Beta-pinene
6	7.28	984	1.24	3-*p*-Menthene
7	7.40	988	0.44	*cis*-Carane
8	7.84	1000	0.68	*trans*-Carane
9	7.95	1004	0.71	Pseudolimonene
10	8.03	1006	1.43	Alpha-phellandrene
11	8.11	1009	1.04	δ-3-Carene
12	8.38	1018	0.48	Alpha-terpinene
13	8.66	1026	21.90	*p*-Cymene
14	8.83	1031	21.66	Limonene
15	8.90	1033	23.13	Beta-phellandrene
16	10.88	1085	0.26	Terpinolene
17	13.07	1140	0.24	Unidentified
18	13.39	1148	0.38	Camphor
19	13.49	1150	1.62	*trans*-Dihydro-alpha-terpineol
20	13.88	1159	0.73	Unidentified
21	14.80	1179	0.39	4-Terpineol
22	15.05	1185	1.98	Cryptone
23	15.42	1192	3.32	Alpha-terpineol
24	18.97	1275	0.53	*p*-Manth-1-en-7-al
25	23.20	1372	1.50	Alpha-copaene

**Figure 1:**
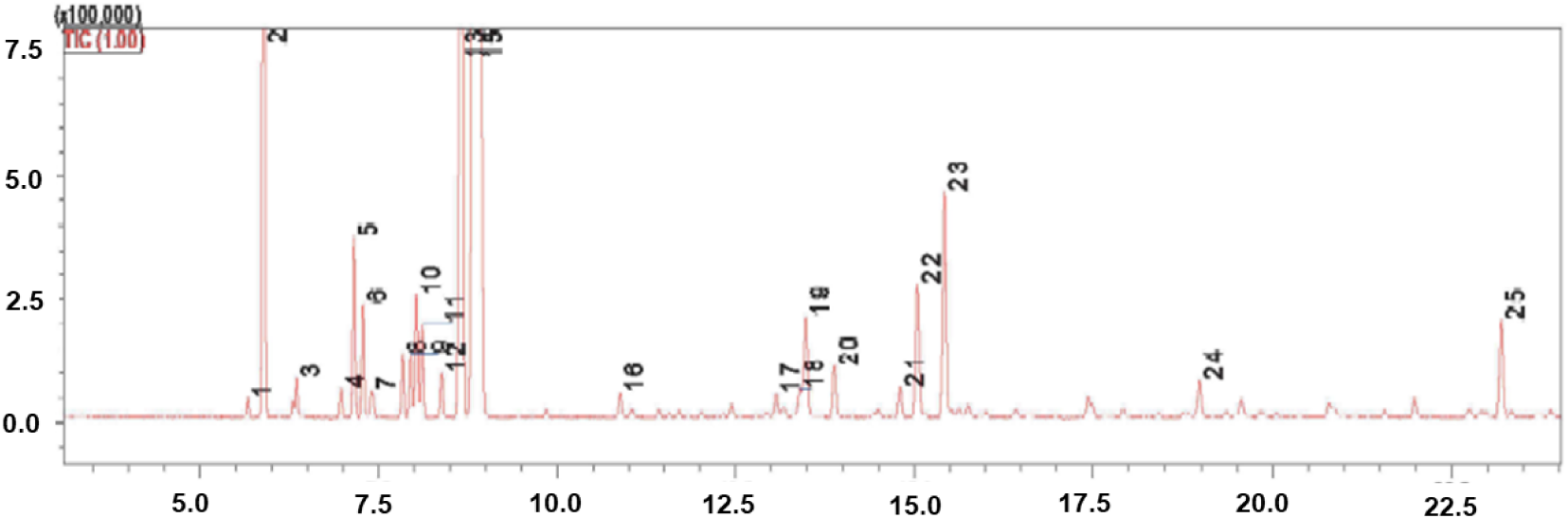
Chromatogram of the *Protium heptaphyllum* essential oil (PHEO) sample obtained by gas chromatography–mass spectrometry-GC-MS analysis performed at the Institute for Research on Natural Products—Federal University of Rio de Janeiro (UFRJ).

### 2.3 Experimental Design

#### 2.3.1 Experiment with Essential Oil of *Protium heptaphyllum* (PHEO)

Juvenile tambaqui (28.23 ± 4.1 g) were randomly assigned to the following treatments: (a) control; (b) vehicle (2 mL of 70% ethanol diluted in 1 L of aquarium water; final concentration 0.14% v/v); (c) PHEO 50 μL·L^–1^; (d) 75 μL·L^–1^; (e) 100 μL·L^–1^; and (f) 150 μL·L^–1^. Fish were exposed to the anesthetic baths for 10 min to evaluate induction, followed by a 5 min recovery period in clean water without PHEO. For all experiments, *n* = 9 refers to nine animals per experimental group. Behavioral assessments, electrocardiographic recordings, opercular rate measurements, and blood glucose determinations were performed using independent biological replicates within each treatment group. The vehicle concentration (0.14% v/v ethanol) did not produce significant differences compared with the water control for behavioral, electrocardiographic, or opercular parameters.

Water quality parameters were monitored throughout the anesthetic exposure period to ensure environmental stability. Temperature, pH, dissolved oxygen, and conductivity were maintained within the recommended physiological range for juvenile *Colossoma macropomum*. Dissolved oxygen levels remained stable during all experimental procedures, minimizing the possibility that cardiac or opercular responses were influenced by environmental factors rather than by PHEO exposure.

#### 2.3.2 Experiment 1—Analysis of Characteristic Behaviors of Anesthetic Induction and Recovery

Considering the contact time for the following PHEO treatments: a) 50 μL·L^–1^, b) 75 μL·L^–1^, c) 100 μL·L^–1^ and d) 125 μL·L^–1^ (*n* = 9/treatment), the latency for the loss of the postural reflex, characterized by lateral recumbency, was evaluated. Subsequently, the animals were removed from contact with PHEO, and the latency for recovery of the postural reflex was assessed. Thirty-six animals were used in this experiment.

#### 2.3.3 Experiment 2—Electrocardiogram (ECG) Analysis

Prior to electrode insertion, fish were anesthetized to minimize stress and movement during the procedure. Electrodes were inserted immediately before the electrocardiographic recording, and the procedure was standardized across all experimental groups to ensure reproducibility and consistency of the recordings. For the analysis and monitoring of cardiac function, the groups were divided as follows: a) Control group; b) Vehicle group; c) Group treated with PHEO 50 μL·L^–1^; d) Group treated with PHEO 75 μL·L^–1^; e) Group treated with PHEO 100 μL·L^–1^; and f) Group treated with PHEO 125 μL·L^–1^ (total of 54 animals). For this purpose, electrodes were made of 925 silver with a diameter of 0.3 mm and a length of 10 mm, and subsequently insulated with liquid insulator. They were made in a nonconjugated manner. The position used for fixing the reference electrode followed the indication of the cardiac vector (negative pole at the base of the heart and positive pole at the apex of the heart). The reference electrode was fixed in the ventral portion of the left opercular opening, 0.2 mm after the end of the opercular cavity. The recording electrode was inserted 2.0 mm from the right opercular aperture. The electrode captured the signal in the vicinity of lead D1. After that, the electrodes were connected to a high-impedance amplifier (Grass Technologies, Model P511) to perform the electrocardiographic recordings. This allowed for the analysis of heart rate (bpm), QRS complex amplitude (mV), QRS complex duration (ms), R–R intervals (ms), P–Q intervals (ms), and Q–T intervals (ms).

#### 2.3.4 Experiment 3—Recording of Opercular Activity during Anesthetic Induction and Recovery

Fish were anesthetized prior to electrode insertion, and electrodes were positioned immediately before the recording session. The procedure followed the same standardization applied in the ECG experiment to ensure methodological consistency among groups. For the analysis of opercular activity, electrodes were made of 925 silver with a diameter of 0.5 mm and a length of 15 mm. The electrodes were made in a conjugated manner at 5 mm and insulated with liquid insulator. The position used for fixing the electrodes and recording the opercular beat was the central part of the right opercular opening. During the recording of the opercular beat, the frequency (beats per minute) and the power of the opercular beat (mV^2^/Hz) were evaluated.

#### 2.3.5 Recording and Analysis of Records

The electrodes were connected to a digital data acquisition system via a high input impedance differential amplifier (Grass Technologies, Model P511), tuned with 0.3 and 300 Hz filtering, with 2000× amplification and monitored with an oscilloscope (ProteK, Model 6510). The recordings were continuously digitized at a rate of 1 kHz on a computer equipped with a data acquisition board (National Instruments, Austin, TX), and were stored on a hard drive and subsequently processed using specialized software (LabVIEW Express).

The analysis of the acquired signals was possible with the aid of a tool built in the Python programming language. The NumPy and SciPy libraries were used for mathematical processing and the Matplotlib library was used for graphing. The graphical interface was developed using the PyQt4 library (Hamoy et al., 2023).

#### 2.3.6 Experiment 4—Blood Glucose Levels in Fish

Blood glucose levels were measured using a portable glucometer (G-TECH FREE, Accumed Produtos Médico Hospitalares Ltda., Brazil). Fish were fasted for 24 h prior to sampling to minimize postprandial glycemic variation. Measurements were performed at two time points: baseline (T0), immediately before exposure to PHEO, and 30 min after exposure (T30). Fish were gently restrained in a moist cloth to reduce handling stress, and approximately 0.5 μL of blood was collected from the caudal vein using a sterile syringe. Glucose determination was performed immediately after collection according to the manufacturer’s instructions. The glucometer was calibrated prior to use following the manufacturer’s quality control recommendations. For each biological replicate (*n* = 9 per group), measurements were performed in duplicate, and the mean value was used for statistical analysis. Although portable glucometers are primarily validated for mammalian blood, they have been widely applied in fish studies as a practical tool for comparative physiological assessment. Nevertheless, potential influences of hematocrit differences on absolute glucose values are acknowledged as a methodological limitation.

## 3 Statistical Analysis

After verifying normality (Kolmogorov–Smirnov test) and homogeneity of variances (Levene’s test), data were analyzed using one-way ANOVA followed by Tukey’s post hoc test. All end points satisfied the assumptions for parametric analysis. Test statistics and corresponding *p*-values are reported in the manuscript (or provided in the Supplementary Material). Statistical analyses were performed using GraphPad Prism 8. Differences were considered statistically significant at *p* < 0.05 (*p* < 0.01 and *p* < 0.001 were considered highly significant).

## 4 Results

The chemical composition of *Protium heptaphyllum* essential oil (PHEO) is presented in Table 1. A total of 25 compounds were identified, predominantly consisting of monoterpenes. The major constituents were β-phellandrene (23.13%), *p*-cymene (21.90%), and limonene (21.66%), which together accounted for the majority of the oil composition. Other relevant compounds included α-pinene (13.60%) and α-terpineol (3.32%), while the remaining constituents were present in lower concentrations (<2%). Overall, the chemical profile of PHEO was characterized by a high proportion of hydrocarbon monoterpenes, with minor contributions from oxygenated monoterpenes and sesquiterpenes, such as α-copaene (1.50%). Two minor peaks remained unidentified, representing less than 1% of the total composition.

Behavioral analysis showed that PHEO caused loss of the postural reflex in fish, and the higher the concentration, the shorter the latency for the onset of the loss of the postural reflex. Fish treated with 50 μL·L^–1^ showed an average loss of postural reflex of 240.8 ± 9.284 s, similar to the group treated with 75 μL·L^–1^ (*p* = 0.265), but superior to the other groups. The group treated with 100 μL·L^–1^ showed an average latency of 175.3 ± 13.72 s, superior to the group treated with 125 μL·L^–1^ (107.1 ± 15.89 s) (Figure 2A).

**Figure 2:**
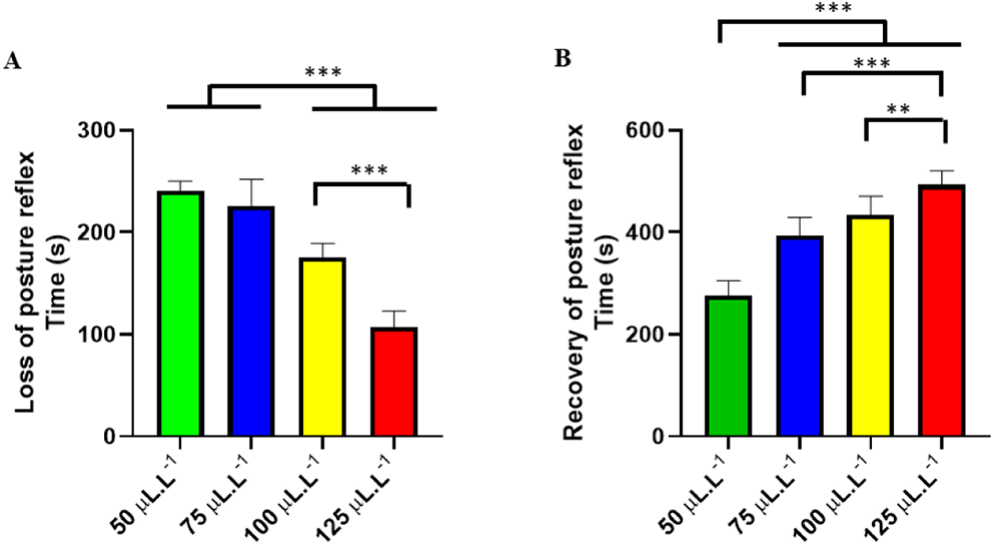
Mean latencies (seconds) for loss of postural reflex during immersion baths with different PHEO treatments (A). Recovery of postural reflex after contact with different concentrations of PHEO (B). (ANOVA followed by Tukey’s test; *p < 0.05, **p < 0.01 and ***p < 0.001).

The recovery of the postural reflex in the group treated with 50 μL·L^–1^ of PHEO occurred in 276.4 ± 28.58 s, which was shorter than the other groups: 75 μL·L^–1^ (397.1 ± 34.98 s), 100 μL·L^–1^ (434.6 ± 36.16 s), and 125 μL·L^–1^ (493.4 ± 27.22 s). All groups showed recovery time dependent on the concentration used; thus, higher concentrations resulted in a longer recovery time for the postural reflex, demonstrating a slower reversibility of the effect for the groups that received higher concentrations (Figure 2B).

The normal electrocardiogram of the tambaqui showed sinus rhythm and an average frequency of 94.00 ± 4.243 bpm. The P wave, the QRS complex, and the T wave could be identified (Figure 2A and B), which was similar to the vehicle group (*p* = 0.999).

These groups presented sinus rhythm, with the presence of all cardiac firings on the electrocardiogram (Figure 3A and B). In 10-s amplification, all ECG elements could be observed: the P wave, which represents atrial contraction; the QRS complex, which represents ventricular contraction; and the T wave, which represents the ventricular repolarization period. From the amplification, it was possible to evaluate the intervals during treatment with PHEO in an immersion bath and their recovery (Figure 3 A–F).

**Figure 3:**
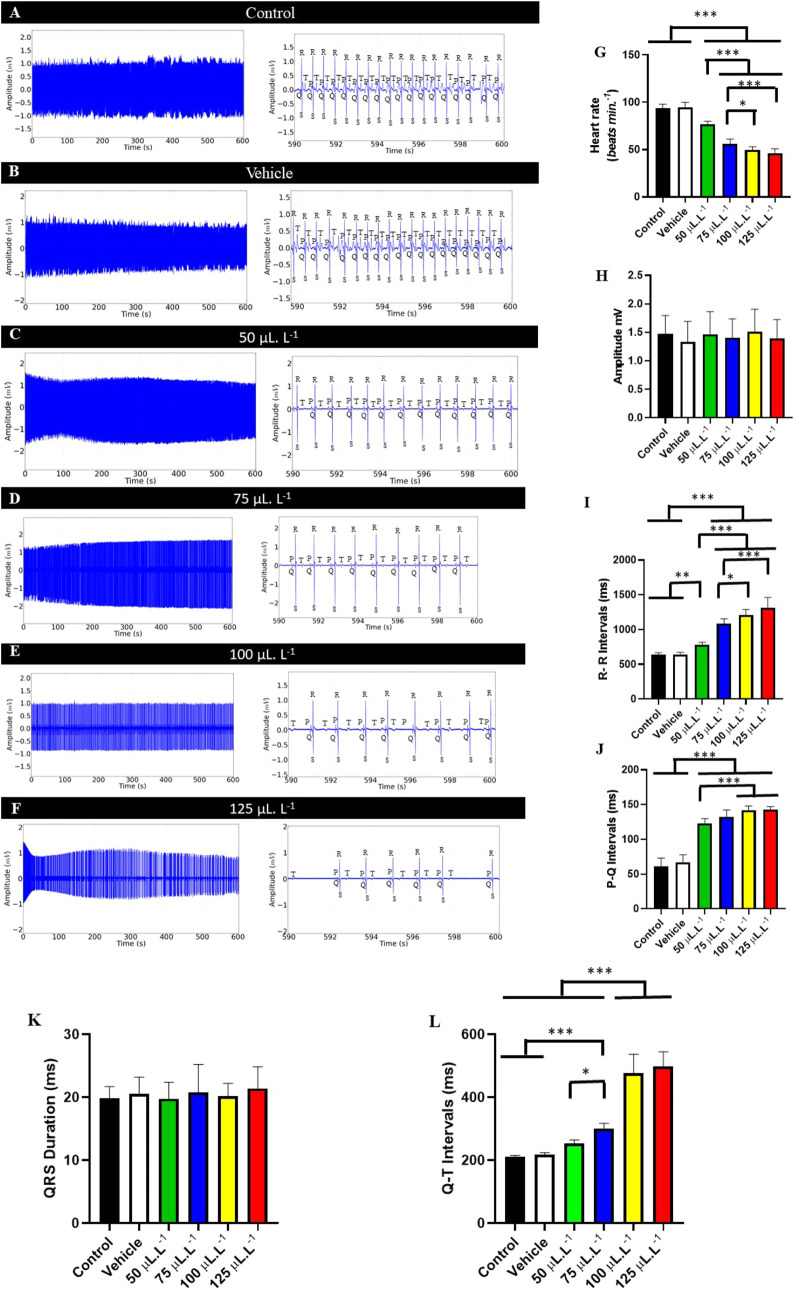
Normal electrocardiographic (ECG) recording of tambaqui, *Colossoma macropomum*, lasting 10 min (left); Enlarged electrocardiogram in the final 10 s of the recording (590–600 s) (center), showing graph elements of the electrocardiogram during anesthetic induction with P wave, QRS complex and T wave and analyzed elements: Heart rate (bpm), QRS complex amplitude (mV), P–Q interval (ms), R–R interval (ms), QRS duration (ms), Q–T interval (ms); for the following groups: Control (A); Vehicle group (B); Group treated with 50 μL L^–1^ (C); Group treated with 75 μL L^–1^ (D); Group treated with 100 μL L^–1^ (E) and Group treated with 125 μL L^–1^ (F). Graph showing mean heart rate (BPM) (G); mean QRS complex amplitude (ms) (H); mean R–R interval (ms) (I); mean P–Q interval (ms) (J); mean QRS complex duration (ms) (K); mean Q–T interval (ms) (L). (ANOVA followed by Tukey’s test; *P < 0.05, **p < 0.01, ***p < 0.001; *n* = 9).

During treatment in an immersion bath with 50 μL·L^–1^ fish showed an 18.43% decrease in heart rate compared to the control group (Figure 3C) for the group treated with 75 μL·L^–1^ of PHEO showed a 40.19% decrease (Figure 3D) for the group treated with 100 μL·L^–1^ showed a 47.04% decrease (Figure 3E) for the group treated with 125 μL·L^–1^ showed a 51.06% decrease (Figure 3F). The treated fish showed bradycardia with maintenance of sinus rhythm, which was intensified by the increase in concentration. The group treated with 125 μL·L^–1^ showed marked arrhythmia (Figure 3F).

Heart rate decreased during treatment with increasing concentrations of PHEO. Controls had a mean of 94.00 ± 4.23 bpm, similar to the vehicle group (94.44 ± 5.63 bpm, *p* = 0.999). However, these rates were higher than the control groups. The group treated with 50 μL·L^–1^ had a mean of 76.67 ± 3.46 bpm, which was higher than the other treated groups. The group treated with 75 μL·L^–1^ (56.22 ± 4.94 bpm) was higher than the groups treated with 100 μL·L^–1^ (49.78 ± 3.38 bpm) and 125 μL·L^–1^ (46.00 ± 5.00 bpm), but the groups treated with 100 μL·L^–1^ and 125 μL·L^–1^ were also higher. L^–1^ were similar (*p* = 0.999) (Figure 3G).

The mean amplitude of the QRS complex in the control group was 1.480 ± 0.316 mV, similar to the other groups (F­(5, 48) = 0.3022; *p* = 0.909) (Figure 3H).

The mean RR interval of the control group was 639.1 ± 27.94 ms, similar to the vehicle group (*p* = 0.999), but lower than the other groups. The group treated with 50 μL·L^–1^ had a mean RR interval of 783.7 ± 36.22 ms, which was lower than the other treated groups. The 75 μL·L^–1^ group (1084.0 ± 71.48 ms) was lower than the groups treated with 100 μL·L^–1^ (1208.0 ± 81.51 ms) and 125 μL·L^–1^ (1316.0 ± 148.1 ms). The groups treated with 100 μL·L^–1^ and 125 μL·L^–1^ were similar (*p* = 0.0573) (Figure 3I).

The mean PQ interval for the control group was 61.22 ± 11.90 ms, showing no difference from the vehicle groups (*P* = 0.7423), but were smaller than the treated groups. The groups treated with 50 μL·L^–1^ (122.4 ± 7.299 ms) were similar to the group treated with 75 μL·L^–1^ (*p* = 0.2149). The group treated with 75 μL·L^–1^ (132.1 ± 10.09 ms) was similar to the groups treated with 100 μL·L^–1^ and 125 μL·L^–1^ (*p* = 0.1665). The groups treated with 100 μL·L^–1^ and 125 μL·L^–1^ were similar (*p* = 0.999) (Figure 3J).

The mean QRS complex duration of the control group was 19.89 ± 1.833 ms, similar to the other groups (F­(5, 48) = 0.3962; *p* = 0.8490) (Figure 3K).

For the control group, the mean QT interval during induction was 210.8 ± 5.142 ms, similar to the vehicle group and the group treated with 50 μL·L^–1^ (*p* = 0.0934). The group treated with 75 μL·L^–1^ (299.6 ± 17.71 ms) was inferior to the groups treated with 100 μL·L^–1^ and 125 μL·L^–1^. The group treated with 100 μL·L^–1^ (476.2 ± 60.72 ms) was similar to the group treated with 125 μL·L^–1^ (*p* = 0.7551) (Figure 3L).

During recovery from exposure to the PHEO concentrations used (50 μL·L^–1^, 75 μL·L^–1^, 100 μL·L^–1^, and 125 μL·L^–1^), reversibility of electrocardiographic changes was observed (Figure 4A–D). However, this reversibility was slower in the groups treated with higher PHEO concentrations.

**Figure 4:**
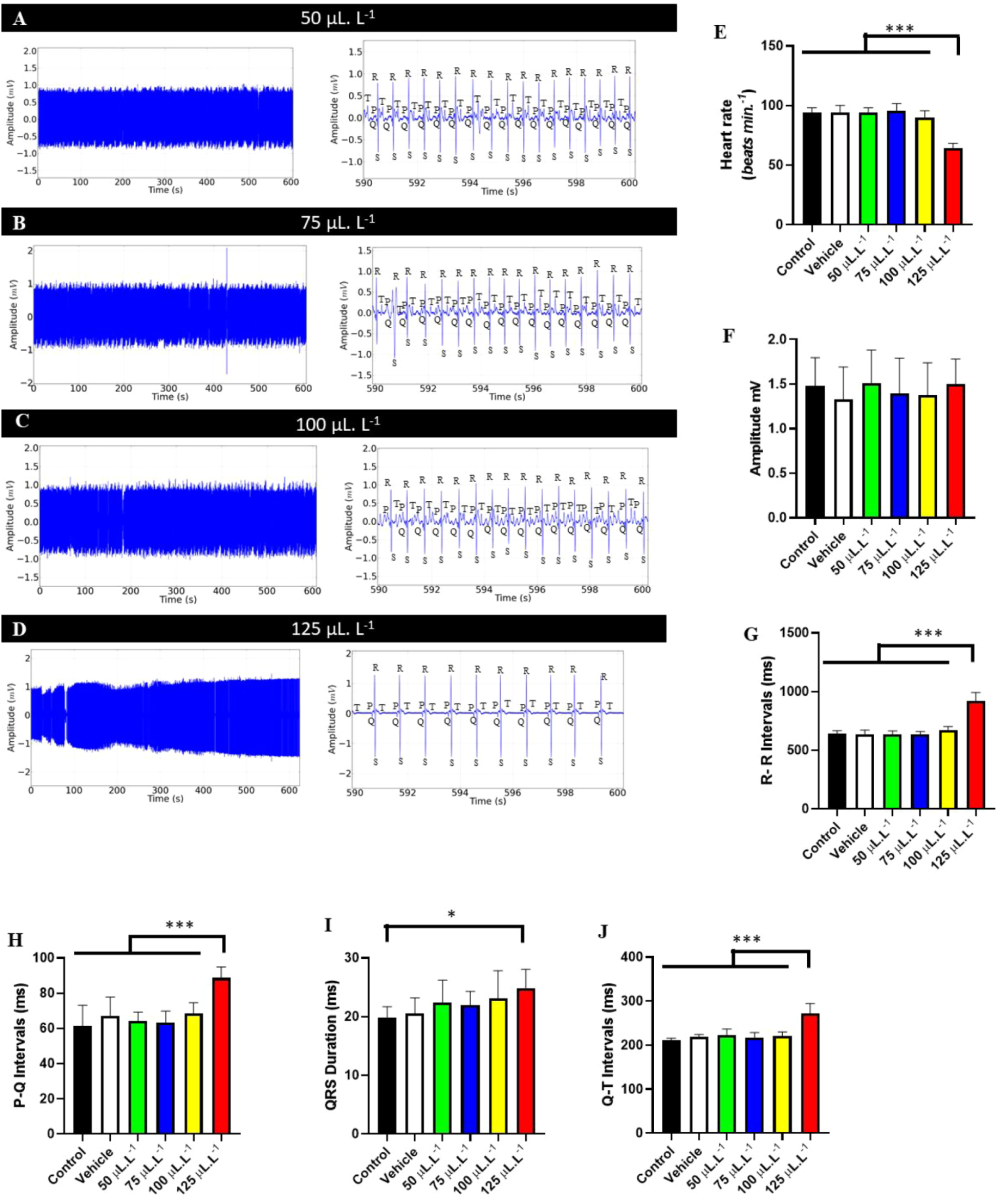
Cardiac activity in juvenile *Colossoma macropomum* during recovery after immersion bathing with different concentrations of PHEO (left). Amplification of the recording in the last 10 s (590–600 s) to identify cardiac triggers (center) during the recovery period after immersion bathing with the following groups: 50 μL·L^–1^ (A), 75 μL·L^–1^ (B), 100 μL·L^–1^, and 125 μL·L^–1^ (D). Graphs showing the mean differences of the ECG graphic elements during recovery: heart rate (bpm) (E); QRS complex amplitude (mV) (F); R–R interval (ms) (G); P–Q interval (ms) (H); QRS complex duration (ms) (I) and Q–T interval (ms) (J). (ANOVA followed by Tukey’s test; *P < 0.05, **p < 0.01, ***p < 0.001; *n* = 9).

During treatment recovery, the groups treated with 50 μL·L^–1^, 75 μL·L^–1^, and 100 μL·L^–1^ were similar to the control group (*p* = 0.4983) (Figure 4A–C). Fish treated with 125 μL·L^–1^ showed 68.68% recovery of cardiac function compared to the control groups (Figure 4D).

During recovery, the control group had a mean heart rate of 94.00 ± 4.24 bpm, similar to the vehicle group, treated with 50 μL·L^–1^, 75 μL·L^–1^ and 100 μL·L^–1^ (*p* = 0.1772). However, they were higher than the group treated with 125 μL·L^–1^ (Figure 4E).

The amplitude of the QRS complex during recovery for the control group was 19.89 ± 1.833 mV, similar to the other groups (F­(5, 48) = 2.814, *p* = 0.0262) (Figure 4F).

The mean RR interval during recovery for the control group was 639.1 ± 27.94 ms, similar to the vehicle groups, groups treated with 50 μL·L^–1^, and 75 μL·L^–1^ and 100 μL·L^–1^ (*p* = 0.4131). The groups treated with 125 μL·L^–1^ (920.8 ± 72.28 ms) were superior to the other groups (Figure 4G).

The PQ interval during recovery in the control group was 61.22 ± 11.90 ms, similar to the vehicle group and the groups treated with 50 μL·L^–1^, 75 μL·L^–1^, and 100 μL·L^–1^ (*p* = 0.4019). The group treated with 125 μL·L^–1^ had a mean of 88.67 ± 6.18 ms, which was superior to the other groups (Figure 4H).

The duration of the QRS complex during recovery in the control group had a mean (19.89 ± 1.83 ms), similar to the vehicle group and the groups treated with 50 μL·L^–1^, 75 μL·L^–1^, and 100 μL·L^–1^ (*p* = 0.2927). The group treated with 125 μL·L^–1^ (24.89 ± 3.18 ms) was superior to the control group (Figure 4I).

During recovery, the QT interval for the control group was 210.8 ± 5.14 ms, similar to the vehicle group, 50 μL·L^–1^, 75 μL·L^–1^, and 100 μL·L^–1^ groups (*p* = 0.4410). The group treated with 125 μL·L^–1^ (271.4 ± 22.92 ms) was superior to the other groups (Figure 4J).

During treatment with PHEO at concentrations of 50 μL·L^–1^, 75 μL·L^–1^, 100 μL·L^–1^ and 125 μL·L^–1^, the opercular movement frequency (omm) showed a concentration-dependent decrease (Figure 5A–F). The control group had an average of 77.56 ± 5.981 omm, which was similar to the vehicle group (*p* = 0.9998). However, they were higher than the treated groups. The group treated with 50 μL·L^–1^ showed a decrease in opercular beat of 25.09%. For the group treated with 75 μL·L^–1^ (41.55%), treated with 100 μL·L^–1^ (46.41%) and treated with 125 μL·L^–1^ (56.16%).

**Figure 5:**
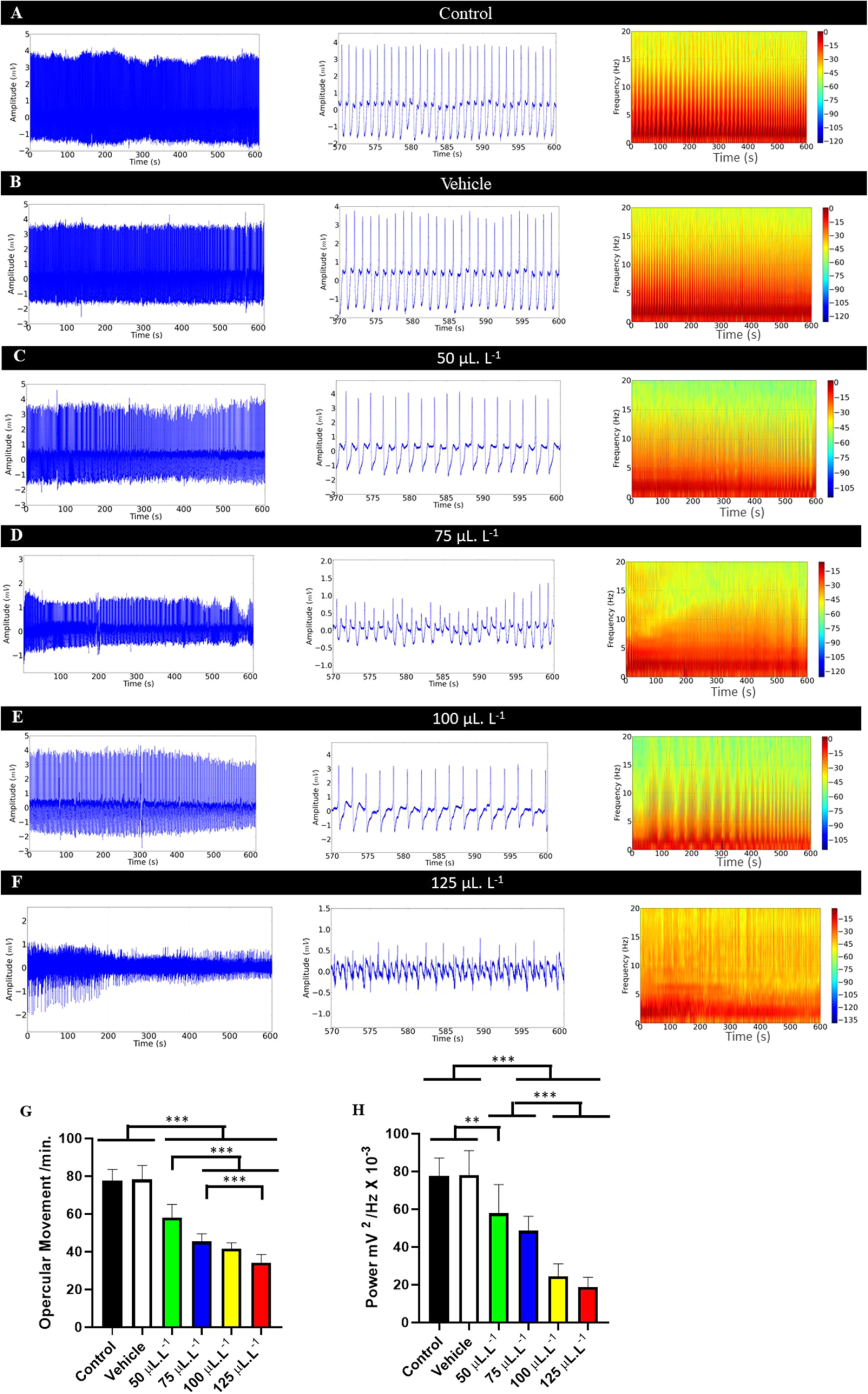
Recording of opercular activity in juvenile *Colossoma macropomum* during immersion bath in different concentrations of PHEO (left). Amplification of the recording in the last 30 s (570–600 s) (center); energy distribution spectrogram during immersion bath at different concentrations of PHEO (right). Verified for the following groups: Control group (A); Vehicle group (B); Group treated with 50 μL·L^–1^ (C); treated with 75 μL·L^–1^ (D); treated with 100 μL·L^–1^ (E) and treated with 125 μL·L^–1^ (F). Presents the average values of opercular movement per minute (omm) (G) and average values of the average power of the opercular movement (mV^2^/Hz) (F). (ANOVA followed by Tukey’s test; *P < 0.05, **p < 0.01, ***p < 0.001; *n* = 9).

The group treated with 50 μL·L^–1^ (58.11 ± 6.90 omm) was superior to the treated groups. The group treated with 75 μL·L^–1^ was similar to the group treated with 100 μL·L^–1^ (*p* = 0.700) and the group treated with 100 μL·L^–1^ (41.56 ± 3.12 omm) was similar to the 125 μL·L^–1^ group (*p* = 0.0605) (Figure 5G).

A decrease in power in the opercular movement recordings (omm) was observed during anesthetic induction. The control group showed an average power of 77.45 ± 9.60 mV^2^/Hz × 10^–^³, which was similar to the vehicle group (*p* = 0.999), but higher than the other treated groups. The group treated with 50 μL·L^–1^ showed a mean (57.90 ± 15.15 mV^2^/Hz × 10^–^³) similar to the group treated with 75 μL·L^–1^ (*p* = 0.3768). The group treated with 100 μL·L^–1^ (24.58 ± 6.522 mV^2^/Hz × 10^–^³) was similar to the group treated with 125 μL·L^–1^ (*p* = 0.8336) (Figure 5H).

During the anesthesia recovery period, the frequency of opercular movement (omm) showed concentration-dependent recovery (Figure 6A–D). The control group had an average of 77.56 ± 5.98 omm, which was similar to the vehicle group, treated with 50 μL·L^–1^, treated with 75 μL·L^–1^, and treated with 100 μL·L^–1^ (*p* = 0.1876). The group treated with 125 μL·L^–1^ (66.67 ± 4.00 omm) was lower than the other groups (Figure 6E).

**Figure 6:**
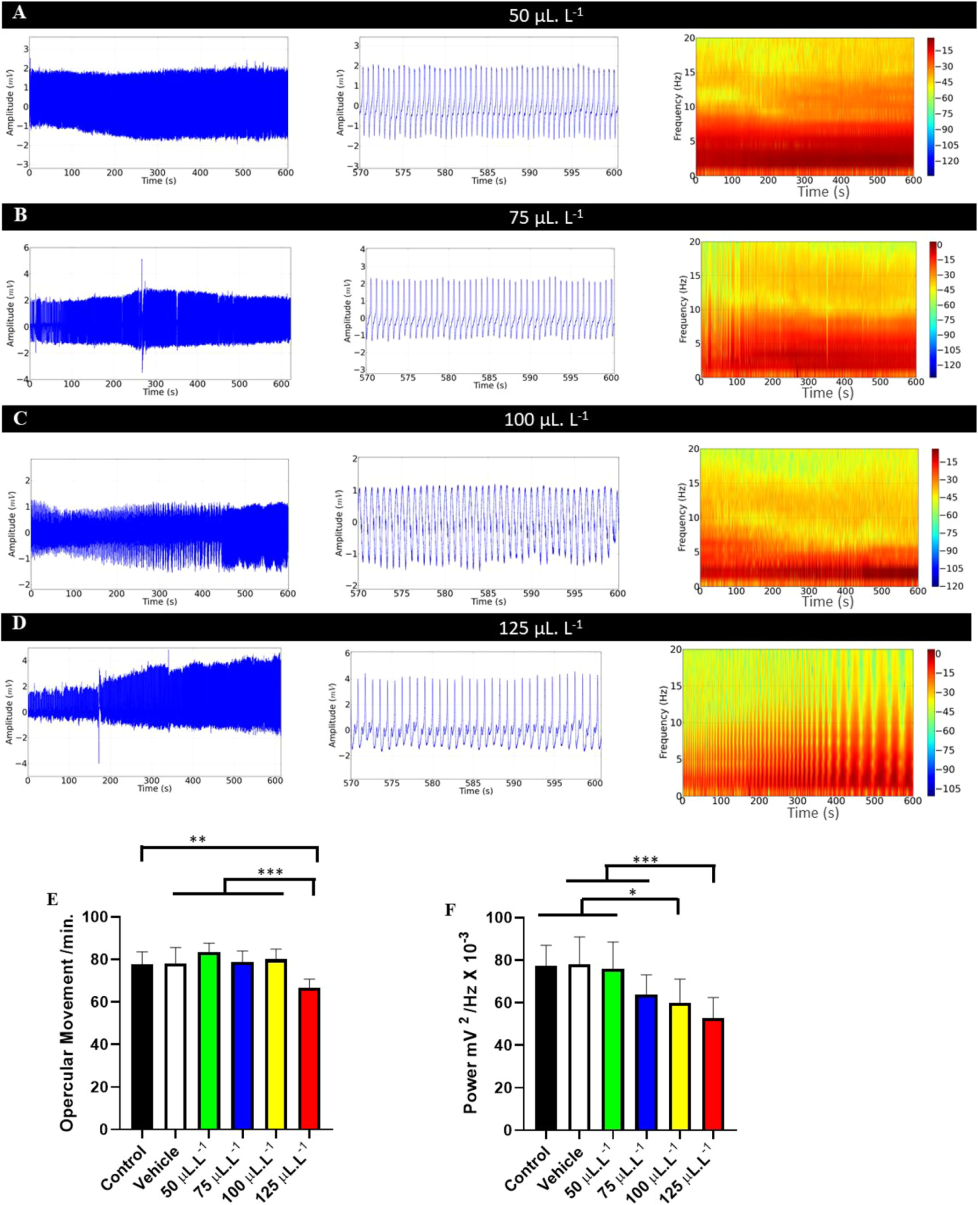
Recording of opercular movement in juvenile *Colossoma macropomum* during recovery after immersion bath with different concentrations of PHEO (left). Amplification of the recording in the last 30 s (570–600 s) for identification of the recordings (center), spectrogram indicating the energy level during animal recovery (right). After immersion bath in the following concentrations: 50 μL·L^–1^ (A), 75 μL·L^–1^ (B), 100 μL·L^–1^ (C), and 125 μL·L^–1^ (D). Average values of opercular movement per minute (E); average values of opercular movement power (mV^2^/Hz) (F). (ANOVA followed by Tukey’s test; *P < 0.05, **p < 0.01, ***p < 0.001; *n* = 9).

An increase in power in the opercular movement (omm) recordings was observed during anesthetic recovery. The control group had an average power of 77.45 ± 9.60 mV^2^/Hz × 10^–^³, which was similar to the vehicle recovery groups, treated with 50 μL·L^–1^ and 75 μL·L^–1^ (*p* = 0.1130). The group treated with 75 μL·L^–1^ was similar to the group treated with 100 μL·L^–1^ and 125 μL·L^–1^ (*p* = 0.2637) (Figure 6F).

The assessment of plasma glucose in the control group (61.56 ± 7.60 mg/dL) was similar to the vehicle T0 group (*p* = 0.9999) and the vehicle T30 group (0.1567), as was the T0 period for all groups (*p* = 0.1868). For the group treated with 50 μL L^–1^, T30 was 94.22 ± 12.756 mg/dL, and 75 μL L^–1^ T30 were similar (*p* = 0.999). However, they were lower than the groups treated with 100 and 125 μL L^–1^. The group treated with 125 μL L^–1^ had a mean T30 of 149.9 ± 22.41 mg/dL, which was higher than the other treated groups (Figure 7).

**Figure 7:**
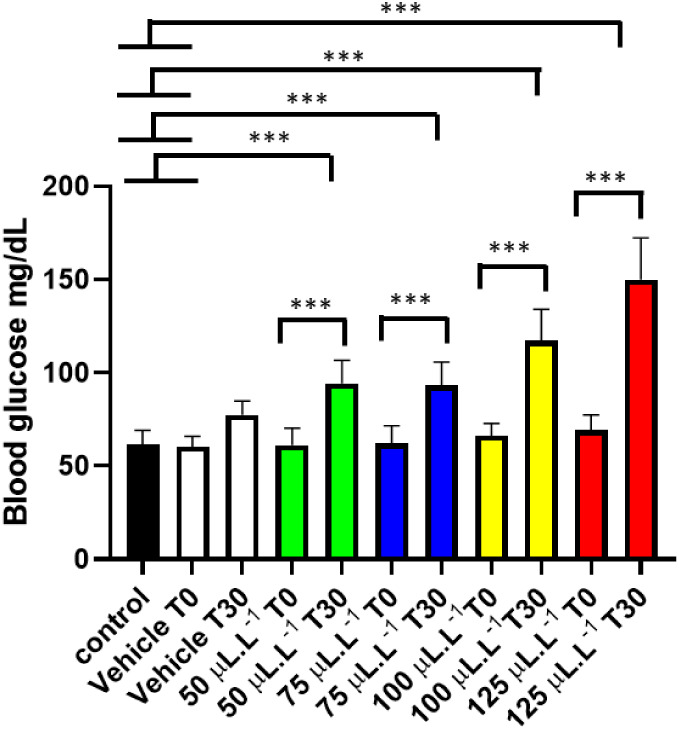
Evaluation of mean blood glucose levels in juvenile *Colossoma macropomum* before immersion in different concentrations of PHEO (time zero T0) and 30 min after immersion (T30) following treatment with PHEO. (ANOVA followed by Tukey’s test; ***p < 0.001; *n* = 9).

## 5 Discussion

The main objective of this study was to evaluate the sedative and anesthetic effects of *Protium heptaphyllum* essential oil (PHEO) in juvenile *Colossoma macropomum* (tambaqui) fish, using an integrated approach that included behavioral, electrophysiological, and physiological parameter analysis, such as plasma glucose. In addition, the chemical composition of PHEO was characterized by gas chromatography, aiming to understand its relationship with the effects observed in the fish.

The electrophysiological results of this study revealed significant effects of *Protium heptaphyllum* essential oil (PHEO) on the cardiac function of juvenile *Colossoma macropomum* fish, highlighting its potential application as an anesthetic and sedative agent in fish. During exposure to PHEO, a concentration-dependent reduction in heart rate (bpm) was observed, indicating bradycardia in all treated groups. In the control group, the mean heart rate was 94.00 ± 4.24 bpm, while in the group treated with the highest concentration of PHEO (125 μL·L^–1^), there was a reduction of up to 51.06% in heart rate, resulting in a mean of 46.00 ± 5.00 bpm. This progressive reduction reflects the direct impact of the bioactive compounds of PHEO on the cardiovascular system, probably associated with the modulation of autonomic activity.

The cardiovascular effects observed in the present study are consistent with previous reports on the anesthetic properties of essential oils in fish. In particular, a recent study conducted in silver catfish (*Rhamdia quelen*) demonstrated that *Protium heptaphyllum* essential oil also induced a concentration-dependent reduction in heart rate during anesthetic induction, followed by gradual recovery after exposure.^22^ Similarly, in the present study, juvenile *Colossoma macropomum* exhibited marked bradycardia during immersion in PHEO, with partial reversibility during recovery, especially at moderate concentrations. Although the overall pattern of cardiac depression was comparable between species, differences in the magnitude of heart rate reduction and recovery kinetics may reflect species-specific physiological characteristics, including metabolic rate, cardiac autonomic regulation, and differential sensitivity to monoterpenes. These findings reinforce the reproducibility of the cardiodepressant effect of PHEO across teleost species while highlighting the importance of defining species-specific therapeutic windows for safe anesthetic application.

The behavioral results observed, with a reduction in the latency time for loss of the postural reflex at increasing concentrations of PHEO, corroborate the inhibitory effects on locomotor activity, typical of central sedatives. The association of these data with electrocardiographic changes, especially bradycardia and prolongation of the RR and QT intervals, suggests that PHEO acts not only through central mechanisms, but also on the autonomic modulation of the cardiovascular system. This convergence of evidence strengthens the hypothesis that compounds such as *p*-cymene and limonene exert synergistic actions on ion channels and neurotransmitters involved in sedation and anesthesia.^23^

The mechanisms underlying the sedative and anesthetic effects of PHEO were not directly investigated in the present study and should therefore be interpreted cautiously. However, previous reports indicate that monoterpenes such as α-pinene and limonene can modulate neuronal excitability through interactions with voltage-gated Na^+^, Ca^2+^, and K^+^ channels, as well as through positive modulation of GABAa receptors, resulting in enhanced chloride influx and neuronal hyperpolarization.^2,4,24^ Based on this evidence, it is reasonable to propose that the observed bradycardia and reduction in opercular activity may involve central and/or autonomic modulation of excitatory pathways. Further mechanistic studies are required to confirm these effects in *Colossoma macropomum*.

Blood glucose is a well-established secondary indicator of stress in fish, reflecting activation of the hypothalamic–pituitary–interrenal (HPI) axis and cortisol-mediated metabolic adjustments. In the present study, PHEO administered within the defined therapeutic window did not induce significant hyperglycemia, indicating that effective sedation was achieved without triggering a pronounced acute stress response. This finding is particularly relevant in aquaculture contexts, where minimizing metabolic disturbance during handling is essential. Although higher concentrations produced cardiophysiological alterations and delayed recovery, glucose levels did not indicate severe metabolic dysregulation within the experimental time frame. Together, these results suggest that PHEO, when applied within the recommended concentration range, provides anesthetic efficacy with limited endocrine-metabolic disturbance in juvenile *Colossoma macropomum*.

It should be noted, however, that the portable G-TECH glucometer employed in this study is validated for mammalian blood. While such devices are frequently used in ichthyological research due to their practicality, differences in hematocrit and erythrocyte morphology between fish and mammals may influence absolute glucose measurements. Therefore, absolute values should be interpreted with caution. Nevertheless, because all experimental groups were assessed under identical methodological conditions, comparative analyses among treatments remain reliable.

In addition, it is plausible to suggest that the observed effects are associated with specific molecular mechanisms, like those of other natural compounds with sedative properties. The major monoterpenes present in PHEO, such as β-phellandrene, *p*-cymene, limonene, and α-pinene, can interact with potassium (K^+^), calcium (Ca^2+^), and sodium (Na^+^) channels, modifying cellular excitability in both neurons and cardiac myocytes. Additionally, these compounds have been associated with the activation of GABAA receptors, which play a central role in regulating neuronal excitability and inducing sedative states. Binding to these receptors can increase the influx of chloride ions (Cl^–^), promoting membrane hyperpolarization and consequent depression of neuronal and autonomic activity. These effects would explain not only the decrease in motor activity but also the bradycardia and reduced opercular movement observed in this study. However, further pharmacological studies are needed to confirm these mechanisms and elucidate the molecular pathways involved.

Although the treated fish exhibited bradycardia, sinus rhythm was maintained in most groups, suggesting that PHEO, at the tested concentrations, did not disrupt the normal electrical conduction of the heart. However, at higher concentrations (125 μL·L^–1^), marked arrhythmias occurred, indicating that increasing the dosage may trigger significant adverse effects on cardiac stability. Cardiac intervals (R–R, P–Q, and Q–T) were altered in a concentration-dependent manner. The R–R interval increased significantly with higher PHEO concentrations, especially in the group treated with 125 μL·L^–1^ (1316.0 ± 148.1 ms), reflecting the reduction in heart rate. The P–Q interval also showed a significant increase at higher concentrations, suggesting a prolongation of atrioventricular conduction. The Q–T interval, which represents the duration of ventricular depolarization and repolarization, was significantly longer in the groups treated with 100 μL·L^–1^ and 125 μL·L^–1^, indicating potential prolongation of ventricular repolarization.

During recovery, electrocardiographic parameters gradually returned to baseline values in the groups treated with lower concentrations of PHEO (50 μL·L^–1^ and 75 μL·L^–1^), demonstrating the reversibility of the cardiac effects. However, in the group treated with 125 μL·L^–1^, recovery was slower, with only 68.68% of normal cardiac function restored at the end of the observation period. These findings indicate that PHEO exerts concentration-dependent and partially reversible cardiac effects, with a narrow therapeutic window. The observed bradycardia may be beneficial in the context of anesthesia and sedation for fish handling, reducing metabolic stress during procedures.

Based on the integrated behavioral and electrophysiological findings, a concentration-dependent therapeutic window can be proposed for PHEO in juvenile *Colossoma macropomum*. Concentrations between 50 and 100 μL·L^–1^ produced effective sedation and anesthesia, characterized by loss of postural reflex, controlled bradycardia with maintenance of sinus rhythm, reduction in opercular frequency, and complete or near-complete recovery within the observation period. In contrast, exposure to 125 μL·L^–1^ resulted in marked arrhythmia, prolonged QT and RR intervals, delayed recovery of cardiac parameters, and elevated plasma glucose levels, indicating increased physiological stress and potential cardiotoxic risk. These findings suggest that concentrations above 100 μL·L^–1^ exceed the recommended anesthetic window and may compromise cardiovascular stability. Therefore, for practical applications in aquaculture, PHEO should be used within the 50–100 μL·L^–1^ range to balance anesthetic efficacy and physiological safety.

Despite the observed benefits, the study also highlights an important limitation: the use of high concentrations of PHEO (125 μL·L^–1^) induced cardiac arrhythmias and a slower recovery of physiological function, which may represent a risk to the safety of animals in situations of prolonged handling. These findings highlight the need to define a safe therapeutic window, where the desired effects of sedation and anesthesia are achieved without compromising physiological homeostasis. Further studies evaluating liver toxicity, oxidative stress, and possible cumulative effects are recommended before large-scale application.

The chromatographic analysis performed in this study is essential to understand the chemical composition of *Protium heptaphyllum* essential oil (PHEO) and its relationship with the sedative and anesthetic effects observed in juvenile *Colossoma macropomum*. The chemical profile of *Protium heptaphyllum* essential oil (PHEO) identified in the present study, characterized predominantly by β-phellandrene (23.13%), *p*-cymene (21.90%), limonene (21.66%), and α-pinene (13.60%), is generally consistent with previously reported compositions for this species. In the study published in *Fish Physiology and Biochemistry*,^22^ PHEO was also described as being rich in monoterpenes, although variations in the relative abundance of individual constituents were observed. Similar variability has been reported in other phytochemical investigations of *Protium heptaphyllum*, where differences in geographic origin, plant organ used, season of collection, and extraction methodology significantly influenced the proportion of major compounds. Despite quantitative differences, the predominance of bioactive monoterpenes across studies reinforces the reproducibility of the oil’s chemical signature and supports the hypothesis that these constituents are primarily responsible for the sedative and cardiodepressant effects observed in teleost species. Such compositional consistency strengthens the translational relevance of PHEO as a potential natural anesthetic in aquaculture.

In aquaculture, the use of anesthetics and sedatives is essential to ensure animal welfare and efficiency in various management practices, such as transport, weighing, vaccination, and slaughter.^25^ This work presents a significant contribution by exploring PHEO as a natural and sustainable alternative to synthetic anesthetics, with advantages and relevance.^22^ PHEO is extracted from a native plant, representing a sustainable option that can reduce dependence on synthetic chemicals, which often present residual toxicity and environmental impact.^26^ The viability of using essential oils such as PHEO in aquaculture can be an economic differentiator, especially in countries like Brazil, which have extensive biodiversity and access to natural raw materials.^27,28^ The concentration-dependent effects and reversibility observed at lower concentrations show that PHEO can be used in a controlled manner, ensuring safety for both animals and the aquatic environment. This study contributes to the development of safer and more effective sedation and anesthesia protocols in fish, providing a scientific basis for the incorporation of natural bioactive compounds into aquaculture practices.

The use of PHEO as a natural anesthetic in farmed fish represents an important innovation for sustainable aquaculture. In our group’s work comparing it to synthetic anesthetics such as benzocaine^29^ and eugenol,^30^ PHEO presents a lower risk of bioaccumulation, is biodegradable, and can be obtained from renewable sources. Furthermore, being a product of plant origin, it aligns with more ethical and consumer-acceptable production practices. The possibility of using forest byproducts for livestock farming also opens doors to production chains integrated with the socio-biodiversity of the Amazon.

It is important to note that the essential oil used in this study was obtained from a single extraction batch. Batch-to-batch variability was not assessed. Given that essential oil composition may vary according to harvest season, geographical origin, and extraction conditions, variations in phytochemical profile could potentially influence the observed biological effects. Therefore, caution should be exercised when extrapolating these findings, and future studies should evaluate compositional variability and its impact on anesthetic efficacy and safety.

Chromatographic data confirmed that the major components of PHEO possess properties recognized in the literature for exerting relevant pharmacological effects, such as sedative, analgesic, and anxiolytic activities, which may justify the behavioral and electrophysiological results obtained.^31^ *p*-Cymene, for example, is an aromatic hydrocarbon with anti-inflammatory properties and anxiolytic potential,^24^ while limonene and beta-phellandrene are associated with relaxing and anesthetic effects.^32,33^ The robustness of the applied methodology is evidenced by the controlled conditions of the equipment, such as the column temperature, the type of detector (FID), and the precise calibration with chemical standards. The choice of the essential oil extraction method by distillation and steam-distillation also contributed to the integrity and representativeness of the volatile compounds analyzed. Additionally, the precise quantification of the major compounds corroborates the hypothesis that the chemical composition of PHEO is determinant for the observed effects, both in the reduction of motor activity (sedation) and in the decrease of heart rate (anesthesia). This analysis offers a scientific basis for future pharmacological and aquaculture applications, suggesting that the major components of PHEO can be explored as naturals alternatives to conventional chemical anesthetics, promoting greater safety and sustainability. The results emphasize the importance of gas chromatography as an indispensable tool for phytochemical studies, allowing not only the characterization of bioactive substances, but also the correlation between chemical structure and biological function.

## 6 Conclusion

In summary, the essential oil of *Protium heptaphyllum* demonstrated efficacy as a sedative and anesthetic in juvenile *C. macropomum*, especially at concentrations of 50 to 100 μL·L^–1^, with reversible physiological effects and absence of serious cardiac alterations. These characteristics reinforce its potential for use in fish farming management protocols. However, for commercial application, it is recommended to conduct field tests, additional toxicological analyses, and standardization of the essential oil composition, considering seasonal and geographical variations.

## Data Availability

The data sets used and/or analyzed during the current study are available from the corresponding author upon reasonable request.
